# Environmental sustainability in sports clubs: development and preliminary validation of the GreenTennis-SUSCLUB questionnaire

**DOI:** 10.3389/fspor.2026.1987947

**Published:** 2026-10-19

**Authors:** Juan Alfonso García-Roca, María José Maciá-Andreu, María T. Morales-Belando, José Antonio Arranz-Escudero, Cem Tinaz, Grasiela Piuvezam, Eva Vegue-Parra

**Affiliations:** 1 Faculty of Sport, Catholic University of Murcia, Murcia, Spain; 2 Olympic Studies Center, Catholic University of Murcia, Murcia, Spain; 3 Department of International Sport Management, The Hague University of Applied Sciences, The Hague, Netherlands; 4 Department of Public Health, Federal University of Rio Grande do Norte, Natal, Brazil; 5 Faculty of Nursing, Catholic University of Murcia, Murcia, Spain

**Keywords:** corporate environmental management, environmental learning, environmental sustainability assessment, pro-environmental behavior, sport policy, sports management, sustainability practices

## Abstract

**Introduction:**

As environmental sustainability becomes an increasingly important challenge for sport organizations, understanding the psychological determinants of pro-environmental behavior among sport professionals is essential. However, instruments specifically designed for sport settings remain scarce. This study developed and preliminarily evaluated the GreenTennis-SUSCLUB questionnaire to assess sustainability-related psychological and behavioral dimensions in tennis clubs.

**Methods:**

The instrument comprises 34 items in total: 6 sociodemographic items, 25 Likert-scale items covering five theoretical dimensions (Knowledge, Attitudes, Practices, Barriers/Needs, and Self-efficacy/Commitment), and three open-ended questions. Content validity was established by an expert panel (*n* = 10), and the field version was completed by 99 tennis professionals from 28 European countries. Psychometric analyses included exploratory factor analysis, internal consistency (Cronbach's *α* and McDonald's *ω*), and thematic analysis of qualitative responses.

**Results:**

The questionnaire demonstrated excellent content validity (S-CVI/Ave = .931) and satisfactory internal consistency across most dimensions (*α* = .831–.871), except for Barriers/Needs (*α* = .533). Factor analysis supported a four-factor structure, merging attitudes and self-efficacy/commitment. Findings revealed an attitude-behavior gap, with strong pro-environmental attitudes (*M* = 4.01) but lower implementation of sustainable practices (*M* = 3.47), mainly due to organizational, financial, and educational barriers.

**Discussion:**

The GreenTennis-SUSCLUB provides a valid and reliable instrument for assessing sustainability-related psychological and behavioral factors, supporting future research and interventions to promote environmental sustainability in sport organizations.

## 1 Introduction

Environmental sustainability has become increasingly important in international sports policies, driven by agendas that promote responsible practices in the field of physical activity and sport. Organizations such as the International Olympic Committee (IOC), through the Olympic Agenda 2020 + 5, and the United Nations (UN), through the Sustainable Development Goals (SDGs), urge the integration of environmental criteria into the planning, management, and evaluation of sports organizations (1, 2). In the European context, the EU Work Plan for Sport 2021–2024 reinforces this approach by proposing lines of action to reduce the environmental impact of sport and support federations and clubs in adopting sustainable measures (3).

Scientific literature indicates that sustainability in sport is an emerging field that requires specific measurement tools capable of translating institutional recommendations into operational diagnoses in sports facilities and organizations. However, research shows that, although various environmental initiatives have been developed in sport, there is still limited evidence based on standardized instruments that allow for a structured assessment of the degree of environmental commitment and guide continuous improvement processes (4).

In this context, research into environmental sustainability applied to sport has begun to incorporate indicators of environmental knowledge and awareness in sports clubs and organizations. Various studies have shown that the level of knowledge about environmental issues is related to the likelihood of adopting more sustainable behaviors and policies within these entities. In this regard, Thormann and Wicker (5) included measures of environmental consciousness in members of non-profit sports clubs and observed that greater awareness was associated with more pro-environmental behaviors. Similarly, environmental knowledge and attitudes predicted willingness to financially support sustainability measures in grassroots sports organizations (6).

Recent research in sports management and sustainability agrees that there is a lack of specific, validated psychometric tools that can be applied to real-life sports club contexts, especially in local and grassroots structures. In this regard, although specific scales have been designed and validated to assess environmental attitudes in contexts of physical activity and sport in nature, with solid factorial structures and adequate levels of reliability (7), and there are scales focused on environmental attitudes or green practices from the perspective of users or sporting events (8, 9), there are few instruments aimed at professionals responsible for decision-making in clubs that simultaneously integrate knowledge, attitudes, practices, structural barriers, and self-efficacy. This limitation makes it difficult to obtain operational diagnoses that guide sustainable improvement processes over time (10, 11).

In this regard, the attitudes of different sports agents have been identified as a relevant factor in explaining the adoption of pro-environmental behaviors and policies in clubs and organizations. Based on the Theory of Planned Behavior (12), it was found that favorable attitudes towards sustainability, together with subjective norms and perceived control, predicted the intention to engage in environmentally responsible behaviors among members of community sports clubs (13). Among professionals such as athletic trainers, it was observed that, despite moderately positive attitudes, perceived barriers related to lack of time, resources, training, or institutional support persisted (14). Previous studies have consistently shown that favorable attitudes and high levels of environmental awareness do not automatically translate into systematic sustainable practices in sports organizations due to structural, organizational, and governance constraints (11, 15).

Another important aspect is the implementation of sustainable practices in sports organizations, which has led to the development of scales to quantify what these institutions are actually doing in areas such as waste management, energy and water consumption, user mobility, and green purchasing. There are tools (9) that offer a one-dimensional scale of green practices, gathering information from the perspective of users and customers on the extent to which sports entities apply sustainable operational measures. Research showed that the implementation of green practices in outdoor sporting events was associated with better perceived quality, greater satisfaction, and an intention to participate again (8). In this area, however, studies focusing on governance structures show that many initiatives remain isolated and low-intensity, without systematic integration into ordinary management (16).

The difficulties in moving towards more sustainable management models in sports clubs and organizations are related to the presence of structural barriers and unmet needs. Studies on sports facilities and entertainment venues point to a lack of financial resources, specialized personnel, and time as key obstacles to the systematic adoption of environmental policies (17). In grassroots sport, ageing facilities, tight budgets, lack of direct control over infrastructure, and lack of technical and training support are described (18). At the European level, problems of governance and the absence of common methodologies for measuring impacts have been described, as well as the need for tools adapted to small and medium-sized clubs (19). From an organizational perspective, these limitations have been interpreted as the result of structural constraints specific to sports organizations, including dependence on external resources, regulatory frameworks and the dynamics of the institutional environment. The literature on sports management has pointed out that these factors tend to favor the adoption of incremental or symbolic sustainable actions, limiting the ability of clubs to implement profound and systematic transformations in terms of environmental sustainability (10).

Another aspect to consider in research on environmental sustainability applied to sport is perceived self-efficacy and personal commitment, which also play an important role in understanding why some people within sports clubs take an active role in sustainable practices. In studies with community club members, perceived behavioral control is associated with the intention to engage in environmentally friendly behaviors, along with attitudes and subjective norms (6, 13). Models combining attitudes, self-efficacy, and environmental commitment show that these factors predict participation in ecological actions, such as collaborating in campaigns or leading environmental initiatives (20). These results support the inclusion of specific dimensions of self-efficacy and personal commitment in the assessment instruments applied in sports clubs (Figure 1).

**Figure 1:**
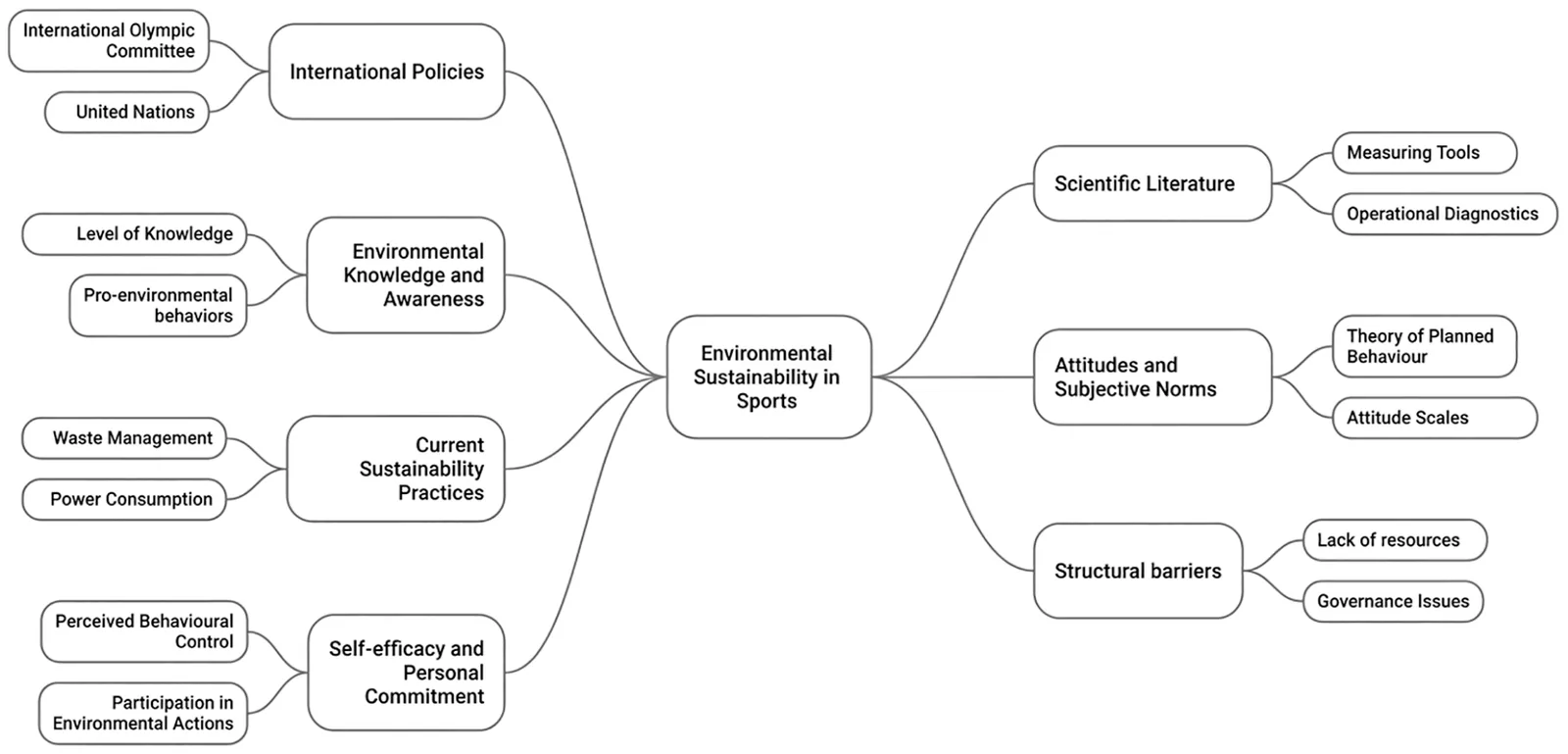
Proposed theoretical model of the GreenTennis-SUSCLUB dimensions. Note. The figure was generated using Napkin.ai (Beta version) on 23 February 2026. This web-based tool (https://www.napkin.ai) was used solely for the visual formatting and layout of the authors’ original conceptual framework and data. No third-party datasets or proprietary materials were used in the output, and the final visual representation was manually verified by the authors to ensure accuracy.

In this context, this study aimed to develop and conduct an initial psychometric evaluation of the GreenTennis-SUSCLUB questionnaire, a context-specific instrument designed to assess environmental sustainability in tennis clubs from the perspective of coaches, managers and tournament directors. More specifically, the study sought to provide initial evidence on the content basis, internal structure, and internal consistency of the instrument as a preliminary step towards its further psychometric consolidation and applied use in sustainability diagnosis within tennis clubs.

## 2 Materials and methods

### 2.1 Design

A quantitative, observational, cross-sectional study was conducted to develop and perform an initial psychometric evaluation of the GreenTennis-SUSCLUB questionnaire for assessing environmental sustainability in tennis clubs. The methodological design followed recommendations for best practices in the development and validation of scales in social and behavioural research, including obtaining preliminary evidence of validity and reliability (21), and was based on the COSMIN framework for the evaluation of measurement properties of item-based instruments, particularly concerning internal structure and internal consistency (22).

### 2.2 Participants

The research team involved in the questionnaire development phase consisted of six university lecturers: five specialists in Sports Science and one psychologist. This team included two tennis experts, a researcher in sports training and performance, a researcher in sports management, and a researcher specialising in education and trainer training. The selection was made through non-probabilistic and intentional sampling, based on a minimum of five years' experience and active roles in this field, both in teaching and research. This group defined the theoretical dimensions of the questionnaire, drew up the initial items, and reviewed their suitability for the context of tennis clubs.

Ten expert judges were selected to evaluate content validity, based on having at least ten years of professional experience as coaches, club managers, or tennis tournament directors. Each judge independently assessed the clarity, consistency, and relevance of the items on a 4-point scale, with 1 being *inadequate* and 4 being *very adequate*.

The final version of the questionnaire, once reviewed by the experts in the first phase and adapted to the native language of each of the participating countries, was subsequently administered to 99 tennis professionals from 28 European countries. This linguistic adaptation was carried out through direct translation by native-speaking members of the consortium in each participating country; a formal forward-backward translation procedure and an independent check of linguistic equivalence across language versions were not applied. This methodological choice is acknowledged as a limitation of the study given its potential impact on content validity and factorial stability across the multilingual versions of the questionnaire. These were selected through intentional non-probability sampling, taking into account the sample inclusion requirements and contact with the experts participating in the project in each of the participating countries. The average age was 48 years (*SD* = 11.9), and the average professional experience in the tennis sector was 29 years. The sample included coaches, club managers, and tournament directors. Participation was voluntary and anonymous via an online form. Table 1 shows the distribution by gender, primary role, type of club, and prior training in sustainability.

**Table 1:** Characteristics of the field study sample (*n* = 99*).*

Variable	Category	*n*	% (*n* = 99)
Gender	Male	66	66.7
	Female	30	30.3
	Prefer not to say	1	1.0
	No response	2	2.0
Primary role in the club	Coach	54	54.5
	Tournament Director	15	15.2
	Manager	14	14.1
	Other	14	14.1
	No response	2	2.0
Type of club	Private	53	53.5
	Community/public	23	23.2
	Municipal	13	13.1
	Schools/University	2	2.0
	Other	6	6.1
	No response	2	2.0
Prior training in sustainability	Yes	41	41.4
	No	47	47.5
	I am not sure	9	9.1
	No response	2	2.0

### 2.3 Instrument

The GreenTennis-SUSCLUB questionnaire includes sociodemographic questions (6 categorical items) and 28 substantive items: 25 closed Likert-type items with a response scale from 1 to 5 and 3 open-ended questions. The 25 Likert items are conceptually organised into five dimensions: 1) Knowledge (*n* = 5); 2) Attitudes (*n* = 5); 3) Current practices (*n* = 5); 4) Barriers/Needs (*n* = 4); and 5) Self-efficacy/Commitment (*n* = 6). The open-ended questions cover perceived barriers, examples of good practices, and opportunities for improvement. The full wording of the 25 Likert items, together with their corresponding variable codes and theoretical dimensions, is provided in Supplementary Table S1. Only the 25 Likert items were used for psychometric analysis, while the categorical and open-ended questions were analysed using descriptive and qualitative procedures, respectively.

### 2.4 Procedure

The development of the GreenTennis-SUSCLUB questionnaire followed an iterative process of construction and initial evaluation, organised into three phases and operationalised through five consecutive versions (v1–v5).

### 2.5 Phase 1. Initial generation and refinement of items (v1–v2)

First, the research team developed an initial bank of items aligned with five theoretical dimensions (Knowledge, Attitudes, Current practices, Barriers/Needs, and Self-efficacy/Commitment). The wording was based on the literature and technical documentation available on environmental sustainability in sport and club management, seeking semantic clarity, absence of redundancies, and suitability for the specific context of tennis clubs. After several internal rounds of review, a first version (v1) with 35 questions was established. Subsequently, as a result of preliminary content refinement, one item was removed due to overlap/conceptual adjustment, resulting in version v2 (34 questions). These figures include sociodemographic profile questions and open-ended questions; the adjustment from v1 to v2 affected one substantive item.

### 2.6 Phase 2. Content validity through expert panel (v2→v3)

Content validity was assessed by a panel of 10 professional experts within the structure of tennis clubs in the areas of training, management, and administration. Each item was assessed according to three criteria: clarity, consistency with the assigned theoretical dimension, and relevance. The evaluation was carried out on a 4-point scale (1 = not adequate; 4 = very adequate). For each item-criterion combination, the Item Content Validity Index (I-CVI) was calculated, defined as the proportion of judges who gave scores of 3 or 4. Following the classic thresholds for panels of this size, I-CVI ≥ 0.78 was adopted as the acceptability criterion; items below the threshold were reviewed based on both the quantitative indicator and the qualitative observations of the panel (23, 24). Based on this evidence, the wording of the affected items was adjusted, and version v3 was consolidated.

### 2.7 Phase 3. Comprehensibility review and field study (v3→v4→v5)

Comprehensibility and apparent validity were verified through an internal cognitive review conducted by five team members who participated in the construction of the instrument, checking the clarity of instructions, the consistency of the response format, the order of blocks, and the absence of operational ambiguities. The questionnaire was then adapted to the consortium's implementation languages (v4) (see Section 2.2 for details on the translation procedure) and administered in electronic format to a sample of 99 tennis coaches and club managers through the project's collaborating entities. The estimated completion time was 12–16 min. The results of the field study were used to finalise the structure and establish the final version v5.

### 2.8 Data analysis

Data analysis was structured on three levels. First, the content validity of the questionnaire was assessed by calculating the Item Content Validity Index (I-CVI) for each of the 25 Likert-type items, considering the criteria of clarity, consistency, and relevance independently. The I-CVI was calculated as the proportion of experts who gave scores of 3 or 4 on a 4-point scale. An I-CVI value ≥ 0.78 was adopted as the acceptability criterion. As an aggregate global indicator, a global S-CVI/Ave was calculated, operationalised as the mean of the I-CVIs of the 25 Likert items in clarity, coherence, and relevance (75 values), excluding categorical and open-ended questions. These calculations were performed using Microsoft Excel 365. Although a total of 99 participants completed the study questionnaire, the sample size analysed per dimension ranged from 95 to 98 (see Table 2) due to item-level missing data. Missing values where not imputed; instead, listwise deletion was applied at the dimension level, including only participants with complete data for all items comprising each respective subscale.

**Table 2:** Descriptive statistics by dimension and internal consistency of the SUSCLUB (1–5 scale).

Dimension	Items	*n*	*M*	*SD*	CI 95% (M)	Cronbach's *α*	McDonald's *ω*	Brief reading
Knowledge	5	97	3.53	0.749	3.37–3.68	0.831	0.835	Good consistency
Attitudes	5	98	4.01	0.658	3.87–4.14	0.856	0.863	High reliability
Current practices	5	95	3.47	0.893	3.29–3.65	0.871	0.873	Excellent reliability
Barriers/needs^a^	4	96	3.49	0.592	3.37–3.61	0.533	0.622	Low-moderate (heterogenoeus)
Self-efficacy/commitment	6	97	3.71	0.605	3.59–3.84	0.819	0.828	Good reliability

M and SD correspond to the mean score for each dimension (1–5). 95% *CIs* were estimated assuming a t-distribution (*df* = N−1). *N* = 99. Sample sizes per dimension (*n* = 95–98) vary due to missing data (unanswered items) in specific scale dimensions. Descriptives were computed using available cases per dimension.

a In Barriers/Needs, the reverse-scored item for access to technical support (bar_accessR) was used to align the direction of the dimension. Given the reliability of this dimension (*α* = .533, *ω* = .622) is below the generally accepted threshold, the mean score reported here should be interpreted with caution and not treated as equivalent in precision to the other, more reliable dimensions.

Secondly, descriptive statistics (mean and standard deviation) were calculated for each dimension of the questionnaire, obtained from the mean scores of its items. Likewise, the internal reliability of the dimensions was estimated using Cronbach's alpha coefficient and, where possible, McDonald's omega coefficient.

Thirdly, the internal structure of the instrument was examined using Exploratory Factor Analysis (EFA) on the 25 ordinal items (Likert 1–5). Given the initial psychometric evaluation stage of the study and the purpose of examining the empirical grouping of items before attempting confirmatory testing, EFA was considered the most appropriate analytic strategy at this stage. This approach was intended to explore the underlying structure of the questionnaire, detect potential overlaps between theoretically related dimensions, and identify items requiring further refinement. Previously, items written in reverse were recoded so that higher values reflected a greater presence of the attribute; in particular, the accessibility item was incorporated in its inverted version (bar_accessR). The adequacy of the data for factor analysis was examined using the KMO index and Bartlett's test of sphericity. Extraction was performed using minimum residuals with Oblimin rotation, and the number of factors was determined by parallel analysis. Given the available sample size and the preliminary nature of this phase, CFA of the five-dimensional theoretical model was not performed at this stage; confirmatory testing was reserved for a subsequent study with a larger and more diverse sample. The analyses were performed in Jamovi 2.6.13.

The responses to the open-ended questions were analysed using thematic analysis, organising the units of meaning into categories related to barriers, good practices, and opportunities for improvement. Pre-processing was performed in Microsoft Excel 365, and coding and thematic organisation in NVivo 14 (QSR International), following an iterative coding procedure and consensus review of the category system (25, 26) (Figure 2).

**Figure 2:**
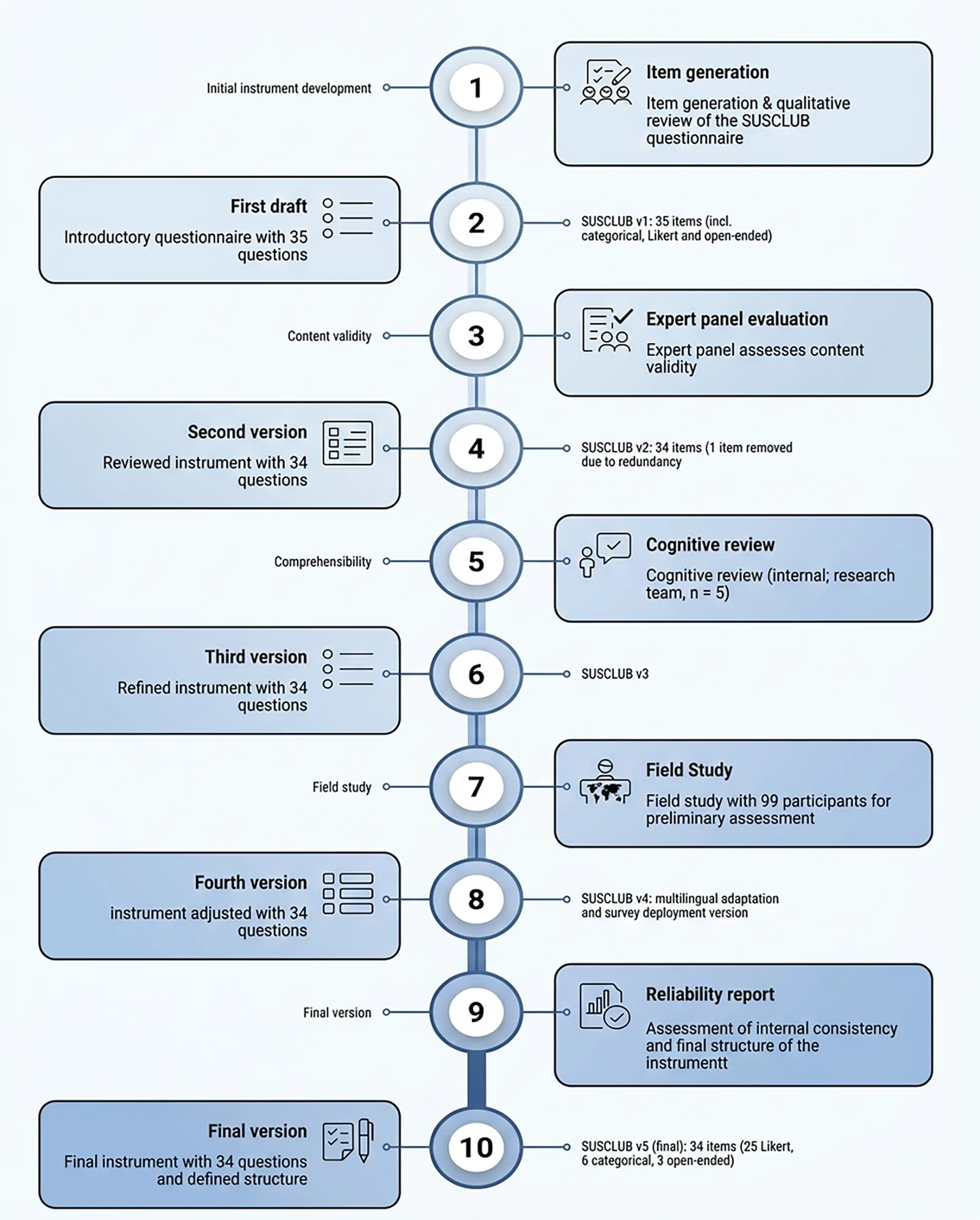
Flowchart of the development and validation process of the SUSCLUB questionnaire. Note. The figure was generated using Napkin.ai (Beta version) on 23 February 2026. This web-based tool (https://www.napkin.ai) was used solely for the visual formatting and layout of the authors’ original conceptual framework and data. No third-party datasets or proprietary materials were used in the output, and the final visual representation was manually verified by the authors to ensure accuracy.

### 2.9 Ethical considerations

The study was approved by the Research Ethics Committee of the Catholic University of Murcia (reference: CE062501, 27/06/2025). Informed consent was obtained from all participants before commencing the questionnaire.

## 3 Results

### 3.1 Content validity

To evaluate content validity, 10 experts assessed the 25 Likert-type items for clarity, coherence, and relevance using a 4-point scale. Following the acceptability criterion (I-CVI ≥ 0.78), 23 of the 25 items reached the threshold across all three criteria. Two items failed to meet the threshold in at least one criterion: one item from the Current practices dimension obtained an I-CVI of 0.70 for relevance, whereas one item from the Barriers/Needs dimension obtained I-CVI values of 0.70 for clarity, coherence, and relevance.

Overall, 71 of the 75 item-criterion evaluations (94.7%) exceeded the established threshold. At the scale level, the overall S-CVI/Ave was 0.931, indicating high content validity across the set of Likert-type items. The two items with lower values were reformulated before the field study, thereby maintaining the dimensional structure of the instrument (Table 2).

### 3.2 Descriptive results and internal consistency of the SUSCLUB dimensions

Means per dimension ranged from 3.47 (Current practices) to 4.01 (Attitudes). Internal consistency values were *α* = 0.831–0.871 and *ω* = 0.835–0.873 for Knowledge, Attitudes, Current practices, and Self-efficacy/Commitment. The Barriers/Needs dimension presented *α* = 0.533 and *ω* = 0.622. Sample size per dimension varied between 95 and 98 due to missing values at the item level (Table 3).

**Table 3:** Items with an I-CVI below the acceptability threshold (expert panel, *n* = 10*).*

Dimension	Item	Criterion with I-CVI < 0.78	I-CVI	Action taken
Current practices	Measures have been adopted to encourage sustainable mobility (e.g., cycling, carpooling)	Relevance	0.70	Reformulated to emphasise proactive promotion rather than mere adoption of measures
Barriers/needs	The club lacks technical staff or expert consultancy to implement environmental initiatives	Clarity; Coherence; Relevance	0.70; 0.70; 0.70	Reformulated to reduce ambiguity and improve conceptual alignment

I-CVI, item-level content validity index; acceptability threshold I-CVI ≥ 0.78.

### 3.3 Structural validity: exploratory factor analysis (EFA)

Structural validity was examined through an EFA on the 25 Likert-type items of the GreenTennis-SUSCLUB. Sampling adequacy was KMO = 0.767, and Bartlett's test of sphericity was significant, χ*^2^*(300) = 1122, *p* < .001. Extraction was performed using minimum residuals with Oblimin rotation; parallel analysis indicated a four-factor solution. Initial values were 6.187 (F1), 2.840 (F2), 1.484 (F3), and 0.778 (F4). Correlations between factors ranged from −0.049 to 0.255 (*r12* = 0.197; *r13* = 0.200; r14 = 0.217; *r23* = 0.255; *r24* = −0.049; *r34* = 0.116).

In the factor loading matrix, the items clustered into four sets: 1) a factor with primary loadings from attitude and self-efficacy/commitment items (e.g., se_collab = 0.844; att_leadbyex = 0.771; se_train = 0.738), 2) a factor with primary loadings from environmental practices (e.g., pr_plastics = 0.802; pr_transport = 0.760; pr_wateruse = 0.735), 3) a factor with primary loadings from knowledge (e.g., kn_bestpract = 0.821; kn_impacts = 0.619; kn_sdgs = 0.576), and 4) a factor with primary loadings from Barriers/Needs (e.g., bar_lacktrain = 0.697; bar_obstacles = 0.633). The reverse-scored item bar_accessR showed a negative loading (−0.424) on the practices factor (Table 4).

**Table 4:** Factor loading matrix (pattern matrix) from the EFA (25 items; 4 factors) and uniquenes*s.*

Item	F1	F2	F3	F4	Uniqueness
se_collab	0.844				0.304
att_leadbyex	0.771				0.320
se_train	0.738				0.433
se_motivate	0.714				0.532
att_reputation	0.684				0.371
att_climate	0.608				0.538
att_policy	0.604				0.545
se_lead	0.541				0.637
att_myrole	0.536		0.331		0.549
se_commit	0.529				0.605
bar_needtools	0.444			0.320	0.651
se_assess	0.398				0.782
pr_plastics		0.802			0.333
pr_transport		0.760			0.427
pr_wateruse		0.735			0.374
pr_waste		0.727			0.435
pr_enwater		0.693			0.477
bar_accessR		−0.424			0.713
kn_bestpract			0.821		0.326
kn_impacts			0.619	0.395	0.433
kn_sdgs			0.576		0.593
kn_resources			0.533		0.598
kn_materials			0.503		0.568
bar_lacktrain				0.697	0.377
bar_obstacles				0.633	0.558

Extraction: minimum residuals; rotation: Oblimin; estimated factor loadings and uniqueness are shown.

### 3.4 Qualitative results from open-ended questions

The thematic analysis identified three main categories: barriers, good practices, and opportunities for improvement. The most frequent barriers were related to financial resources, institutional dependency, and technical training. The good practices described included waste management, efficient water use, and basic energy efficiency measures. Opportunities for improvement were associated with specific training, the availability of operational guides, and internal leadership in sustainability.

## 4 Discussion

This study addresses the development and initial psychometric evaluation of the GreenTennis-SUSCLUB questionnaire, designed as a specific tool to diagnose environmental sustainability in tennis clubs from the perspective of coaches and managers. This approach responds to the need, highlighted in the literature, for standardised and contextually adapted instruments that allow for the translation of regulatory frameworks and institutional recommendations on sustainability into operational diagnostics in grassroots sports organisations (4, 11, 27). In this sense, the results obtained are interpreted in consistency with an initial instrumental approach, aimed at establishing preliminary evidence of validity and reliability, as recommended by methodological guidelines for the progressive development of scales in social and behavioural research (21, 22).

The high agreement observed in content validity, reflected in I-CVI values mostly equal to or greater than 0.78 and an overall S-CVI/Ave of 0.931, is consistent with classical criteria for expert panels and supports the semantic and conceptual adequacy of the items included in the instrument (23, 24, 28). This result suggests that the defined theoretical dimensions—knowledge, attitudes, practices, barriers, and self-efficacy—are correctly represented at the content level, reinforcing the consistency between the proposed conceptual framework and the empirical operationalisation of the construct, as recommended in early-stage instrumental studies (29, 30).

The descriptive pattern observed in the sample of tennis professionals shows relatively high levels of attitudes and self-efficacy/commitment, alongside more moderate levels of current practices and a noticeable presence of perceived barriers. This descriptive profile should nonetheless be interpreted with caution: 41.4% of respondents reported prior training in sustainability, a proportion unlikely to be representative of tennis club managers as a whole, suggesting that the sample may be skewed towards professionals who where already environmentally conscious and motivated to participate; consequently, the attitude and self-efficacy scores reported here may overestimate those of the broader population of club managers. Nevertheless, this profile is congruent with previous studies on sustainability applied to sport, which have consistently pointed to the existence of a gap between favourable predispositions towards sustainability and the effective implementation of environmental measures in sports clubs and organisations (10, 11, 15). From a theoretical perspective, this pattern is compatible with explanatory models such as the Theory of Planned Behaviour, in which behaviour depends not exclusively on attitudes, but also on perceived behavioural control and contextual and organisational constraints (12), findings that have also been reported in community sport club settings (5, 6, 13). In the specific context of European grassroots tennis clubs, this attitude–practice gap may be understood not only as an individual-level inconsistency, but also as a reflection of governance conditions in which favourable views towards sustainability coexist with limited organisational capacity, ageing infrastructure, restricted autonomy, and insufficient technical support for systematic environmental action.

Regarding the internal structure of the instrument, the EFA suggested a four-factor solution. The most relevant finding was the joint clustering of the items intended for Attitudes and Self-efficacy/Commitment, indicating empirical overlap between conceptually close domains in this sample of professionals. This pattern is consistent with the literature on pro-environmental behaviour in organisational contexts, where favourable orientations towards sustainability and perceived control or capability to act often operate in a closely interdependent manner. At the same time, this overlap should not be interpreted as evidence that both constructs are theoretically equivalent. Rather, it suggests that, in this context, valuing sustainability and perceiving oneself as capable of promoting change may be experienced as part of a closely connected disposition towards environmental action in the club setting. From a progressive validation perspective, these results support an interpretable alternative structure without invalidating the initial five-dimension conceptual framework (14, 20).

A particularly relevant issue concerns the behaviour of the Barriers/Needs dimension. In contrast to the other blocks, this dimension showed lower internal consistency and a less clearly defined factorial pattern. This suggests that the construct may be more heterogeneous than initially expected. Given this low reliability (*α* = .533, *ω* = .622), the descriptive mean score obtained for this dimension (*M* = 3.49; Table 2) should be interpreted with caution and not treated as equivalent in precision to the scores of the other, more reliable dimensions. Rather than behaving as a fully homogeneous dimension, the Barriers/Needs block appears to combine at least two closely related but not identical contents: perceived obstacles to implementation, on the one hand, and perceived needs for support, tools, or technical guidance, on the other. This interpretation is reinforced by the behaviour of bar_accessR, which did not align clearly with the remaining barrier items and showed a negative loading on the practices factor. This result may reflect either residual wording effects associated with reverse scoring or a deeper conceptual distinction between perceived barriers and perceived availability of operational support. In either case, the findings suggest that this block requires particular caution in interpretation and should be prioritised in future refinement of the questionnaire. At the same time, its behaviour is substantively informative, as it highlights that environmental sustainability in grassroots tennis clubs is shaped not only by favourable attitudes or declared commitment, but also by the organisational conditions that enable or constrain action in practice.

Finally, the qualitative results derived from the open-ended questions provide more than a simple descriptive complement to the quantitative findings. Rather, they offer convergent support for the interpretive validity of the instrument. The barriers most frequently reported qualitatively—financial limitations, institutional dependency, and insufficient technical training—are consistent with the weaker and more heterogeneous functioning of the Barriers/Needs block. Likewise, the identification of low-intensity environmental practices and opportunities for improvement linked to training, resources, and internal leadership is coherent with the quantitative pattern of relatively favourable attitudes and self-efficacy/commitment combined with more moderate levels of current practices. Taken together, this convergence between quantitative and qualitative evidence strengthens the interpretation that the GreenTennis-SUSCLUB is capturing a meaningful sustainability profile in tennis clubs, while also identifying the domains that remain conceptually or psychometrically less stable and therefore require further refinement, in line with mixed-methods approaches recommended for the study of complex and contextual phenomena in sport organisations (25, 26).

## 5 Limitations

There are limitations that must be considered when interpreting the findings of this study. Firstly, the sample was obtained through non-probability sampling, which restricts the generalisability of the results to all tennis club contexts and types. More specifically, 41.4% of participants reported having previously received training in sustainability, a proportion that is unlikely to reflect club managers in general; this points to a probable self-selection effect, whereby professionals who were already environmentally aware and motivated were more likely to take part, which may have inflated the favourable attitudes and self-efficacy scores reported here and constrains the external validity of the findings. Secondly, the questionnaire is based on self-reporting; therefore, responses may be influenced by social desirability bias and differences in the perception and reporting of sustainable practices according to professional roles and organisational settings. Thirdly, although content validity was supported by experts and internal consistency was adequate for most dimensions, the Barriers/Needs block showed less homogeneous metric performance, suggesting conceptual heterogeneity within that domain. Fourthly, although the data met acceptable criteria for factorability, the sample-to-item ratio (approximately 4:1) is at the borderline of what is generally considered adequate for exploratory factor analysis; this limits confidence in the stability of the four-factor solution and the associated loadings, and the EFA findings should therefore be interpreted as preliminary and sample-specific until replicated in a larger sample. In addition, the cross-national composition of the sample (99 professionals across 28 countries) combined with the multilingual adaptation of the questionnaire is a substantive threat to the comparability of the results: because the translation into each country's language was carried out directly by consortium partners without a formal forward-backward procedure or an independent check of linguistic equivalence, item wording may not be fully equivalent across languages, introducing cultural and semantic noise that could have affected both individual responses and the stability of the exploratory factor solution. This limitation should be considered when interpreting both the psychometric findings and their generalisability across the participating countries. Finally, another limitation of the study relates to the presence of item-level missing data across the questionnaire dimensions. Although the total sample comprised 99 participants, the effective sample size per dimension ranged between 95 and 98 valid cases due to non-response on specific items. However, missingness was minimal (< 5% per subscale) and handled via dimension-level listwise deletion to preserve data integrity, making it unlikely to introduce substantial non-response bias or compromise the statistical validity of the internal consistency metrics.

## 6 Future research directions

Future research should expand the psychometric evidence of the instrument by applying it to larger and more diverse samples of tennis professionals. This would allow for a more robust testing of the five-dimensional theoretical model through CFA and, in parallel, a comparison of its fit against the four-factor solution suggested by the EFA. Furthermore, it is recommended to evaluate measurement invariance across relevant subgroups (e.g., coaches vs. managers, different club types, and geographical contexts) before conducting group comparisons. It is also pertinent to further investigate the functioning of the Barriers/Needs dimension, considering its possible reorganisation into subdomains (e.g., internal vs. external barriers) and/or item refinement to improve internal consistency and structural clarity. Additionally, further evidence of validity should be incorporated, including temporal stability via test–retest, relationships with external sustainability indicators, and sensitivity to change in longitudinal designs following specific interventions in clubs. Finally, strategies for handling missing data should be explored to minimise case loss in latent variable analyses.

## 7 Conclusions

This study describes the development and initial psychometric evaluation of the GreenTennis-SUSCLUB questionnaire for assessing environmental sustainability in tennis clubs from the perspective of coaches and managers. The available evidence supports the adequacy of the instrument's content and acceptable internal consistency in most of its dimensions, whereas the Barriers/Needs block showed less homogeneous performance, while also emerging as a particularly relevant domain for understanding the practical constraints surrounding sustainability implementation in tennis clubs. The internal structure identified through exploratory analysis proved interpretable, although confirmatory verification of the theoretical model could not be established at this stage. Given the sample-to-item ratio obtained in this initial validation, this four-factor structure should be regarded as preliminary and provisional until it is replicated in larger, independent samples. Overall, the findings support the use of GreenTennis-SUSCLUB as a structured tool for initial diagnostic assessment in professional contexts, pending additional confirmatory and longitudinal evidence in subsequent studies.

## Data Availability

The raw data supporting the conclusions of this article will be made available by the authors, without undue reservation.
