# Supplementary material for: Environmental sustainability in sports clubs: development and preliminary validation of the GreenTennis-SUSCLUB questionnaire
Source: Front Sports Act Living. 2026 Oct 19;8:1987947. doi: 10.3389/fspor.2026.1987947 (PMC13724483; doi:10.3389/fspor.2026.1987947)
Supplement: Supplementary file 1 [file Table1.pdf]

Supplementary Table S1

Full wording of the 25 Likert-scale items of the GreenTennis-SUSCLUB questionnaire, by theoretical dimension, with corresponding variable codes (as used in Table 4 of the main text)

Dimension	Item #	Item wording (English)	Variable code
Knowledge	1	I am aware of the main environmental impacts generated by a tennis club.	*kn_impacts*
Knowledge	2	I am familiar with the Sustainable Development Goals (SDGs) as applied to sport.	*kn_sdgs*
Knowledge	3	I know examples of sustainable best practices in sports facilities.	*kn_bestpract*
Knowledge	4	I know which types of sports materials or surfaces have a lower environmental impact.	*kn_materials*
Knowledge	5	I am informed about how sports activities affect the use of natural resources (water, energy, land).	*kn_resources*
Attitudes	6	I believe that tennis clubs should adopt an active environmental sustainability policy.	*att_policy*
Attitudes	7	I am concerned about the impact of climate change on sport.	*att_climate*
Attitudes	8	I believe my professional role can contribute to a shift toward a more sustainable sports model.	*att_myrole*
Attitudes	9	I think tennis clubs should lead by example in matters of environmental sustainability.	*att_leadbyex*
Attitudes	10	I believe that integrating sustainability into the daily operations of the club enhances its image and reputation.	*att_reputation*
Current practices	11	My club implements measures to reduce energy and water consumption.	*pr_enwater*
Current practices	12	Recycling or responsible waste management is promoted within the club.	*pr_waste*
Current practices	13	My club promotes the use of sustainable transportation options such as bicycles, public transit, or carpooling.	*pr_transport*
Current practices	14	Measures have been adopted in my club to reduce the use of single-use plastics.	*pr_plastics*
Current practices	15	My club promotes the efficient use of water on courts, in showers, or in garden areas.	*pr_wateruse*
Barriers/Needs	16	There are obstacles in our club that make it difficult to implement sustainable practices (e.g., lack of resources, resistance to change, unclear regulations).	*bar_obstacles*
Barriers/Needs	17	I believe there is a lack of specific training on sustainability in sport.	*bar_lacktrain*
Barriers/Needs	18	It would be helpful to have practical tools (e.g., checklists, protocols, indicators) to implement sustainable improvements in the club.	*bar_needtools*
Barriers/Needs	19	My club has access to technical staff or expert guidance to support environmental initiatives.	*bar_accessR*
Self-efficacy/Commitment	20	I feel capable of leading or proposing sustainable changes in my club.	*se_lead*
Self-efficacy/Commitment	21	I am willing to receive training or participate in initiatives that promote sustainability in sport.	*se_train*
Self-efficacy/Commitment	22	My personal commitment to sustainability influences my professional decisions.	*se_commit*
Self-efficacy/Commitment	23	I am in a position to motivate other club members toward more sustainable management.	*se_motivate*
Self-efficacy/Commitment	24	I am willing to collaborate with other clubs or organizations to share sustainable initiatives.	*se_collab*
Self-efficacy/Commitment	25	I feel able to assess whether a practice in the club is environmentally friendly or not.	*se_assess*
